# Freehand Technique for Pedicle Screw Placement during Surgery for Adolescent Idiopathic Scoliosis Is Associated with Less Ionizing Radiation Compared to Intraoperative Navigation

**DOI:** 10.3390/jpm14020142

**Published:** 2024-01-27

**Authors:** Peter Obid, Sebastian Zahnreich, Andreas Frodl, Tamim Rahim, Thomas Niemeyer, Moritz Mayr

**Affiliations:** 1 Department of Orthopaedics and Traumatology, University Medical Center Freiburg, 79106 Freiburg, Germany; andreas.frodl@uniklinik-freiburg.de (A.F.); moritz.mayr@uniklinik-freiburg.de (M.M.); 2 Department of Radiation Oncology and Radiation Therapy, Mainz University Hospital, 55131 Mainz, Germany; zahnreic@uni-mainz.de; 3 Spine and Scoliosis Center, Asklepios Klinik Wiesbaden, 65197 Wiesbaden, Germany; t.rahim@asklepios.com (T.R.); t.niemeyer@asklepios.com (T.N.)

**Keywords:** intraoperative navigation, freehand technique, pedicle screws, adolescent idiopathic scoliosis, radiation exposure, cancer risk, complications, safety

## Abstract

Purpose: We aim to compare radiation exposure and implant-related complications of the freehand (FH) technique versus intraoperative image-guided navigation (IN) for pedicle screw placement in adolescent idiopathic scoliosis (AIS) and estimate associated lifetime attributable cancer risks. Methods: A retrospective analysis of prospectively collected data from 40 consecutive AIS patients treated with pedicle screw instrumentation using the FH technique was performed. The dose area product (DAP) and effective dose (ED) were calculated. Screw-related complications were analysed, and the age- and gender-specific lifetime attributable cancer risks were estimated. The results were compared to previously published data on IN used during surgery for AIS. Results: There were no implant-related complications in our cohort. Implant density was 86.6%. The mean Cobb angle of the main curve was 75.2° (SD ± 17.7) preoperatively and 27.7° (SD ± 10.8) postoperatively. The mean ED of our cohort and published data for the FH technique was significantly lower compared to published data on the IN technique (*p* < 0.001). The risk for radiogenic cancer for our FH technique AIS cohort was 0.0014% for male patients and 0.0029% for female patients. Corresponding risks for IN were significantly higher (*p* < 0.001), ranging from 0.0071 to 0.124% and from 0.0144 to 0.253% for male and female patients, respectively. Conclusion: The routine use of intraoperative navigation in AIS surgery does not necessarily reduce implant-related complications but may increase radiation exposure to the patient.

## 1. Introduction

First introduced by Suk et al. in 1995, the use of thoracic pedicle screws for posterior correction and fusion (PSF) has become the gold standard for the surgical treatment of adolescent idiopathic scoliosis (AIS) [1]. Segmental pedicle screw constructs allow the application of high correction forces and result in a good long-term outcome [2,3,4,5]. The accurate placement of pedicle screws is paramount since potential risks of misplaced pedicle screws include neurological or vascular complications [6,7]. The freehand (FH) technique for the implantation of pedicle screws has been proven to be effective and safe for the correction of scoliosis [8,9]. Since the introduction of navigation techniques in spine surgery, the implantation of pedicle screws using image-guided navigation (IN) has become more and more popular. An increased accuracy of pedicle screw placement using IN techniques has been described in several studies [10,11]. The use of IN techniques has also been introduced into scoliosis surgery [12,13]. However, all IN techniques have been shown to be associated with an increased radiation dose for the patient [14,15,16]. Any additional radiation dose administered during IN can have serious consequences for the patient and increase the risk of developing late sequelae, with radiation-induced malignancies being one of the most detrimental outcomes [17]. In particular, radiation exposure at an early age immensely increases the risk of radiogenic late consequences compared to exposures during adulthood. AIS patients are already exposed to additional higher levels of radiation due to repeated radiological examinations throughout their course of treatment [18,19,20,21].

Therefore, the risk–benefit assessment should be clearly in favor of a significantly higher clinical benefit for IN compared to the FH technique in AIS surgery. However, to date, no studies have compared implant-related complications and radiogenic cancer risk between the FH technique and IN during surgery in AIS patients. This study aims to provide a further understanding of the surgical and radiobiological implications of the use of IN during surgery for AIS compared to the FH technique.

## 2. Materials and Methods

Radiation exposure and radiogenic cancer risk: We retrospectively reviewed prospectively collected data of 40 consecutive patients (2 males and 38 females) at a single center that underwent PSF for AIS using the FH technique. Due to concerns about radiation exposure, we did not create a control group.

Patients with an etiology other than idiopathic scoliosis, older than 18 years, and a follow-up of less than two years were excluded. Basic demographic data, radiation exposure, and implant-related complications were analyzed. The patients’ intraoperative radiation exposure was obtained as dose area product (DAP), representing the absorbed dose multiplied by the exposed area (Gy × cm^2^). For a conservative estimation of the radiogenic cancer risk, the DAP was converted into an effective dose (ED) (mSv) according to models based on the ICRP (International Commission on Radiological Protection) publication 103 and UNSCEAR 2006 report for anterior–posterior and lateral thoracic spine projections using a conversion factor of 0.24 mSv × Gy^−1^ × cm^−2^ [21,22]. For the calculation of the ED from a dose length product (DLP), a conversion factor for lumbar spine examinations of 0.169 mSv × mGy^−1^ × cm^−1^ was applied according to Lee et al. [23]. The total lifetime cancer risk was determined according to Wall et al. using specific risk coefficients for cancer incidence per unit ED (% per Sv) as a function of age at exposure and sex [22]. For a comprehensive comparison of the radiation exposure and associated radiogenic cancer risk between the FH technique and IN for the treatment of AIS, we reviewed and included all the relevant literature, providing necessary radiation dose information from which we calculated the estimated lifetime attributable cancer risk. For this purpose, conversion factors for anterior–posterior and lateral thoracic spine examinations were applied for the age group of 10–19 years (male: 6.4, female: 13) or the age group of 20–29 years (male: 5.2, female: 11). We performed a comprehensive comparison of the determined ED and radiogenic cancer risk from our patient cohort with previously published studies on the use of the FH technique or IN (see Table 1). A literature search was conducted on Pubmed. The search was based on “(idiopathic scoliosis) AND (navigation)” (All Fields). Other keywords utilized were “scoliosis”, “intraoperative navigation”, “complications”, “radiation exposure”, “effective dose”, and “cancer risk”.

The purpose of the present study is to compare radiation exposure and associated cancer risk of IN and FH techniques for the surgical treatment of AIS. However, since most studies were found to embrace a very heterogeneous patient collective, we decided to include studies dealing with etiologies other than idiopathic scoliosis. Non-comparative studies that were otherwise deemed relevant were also included.

**Figure 1:**
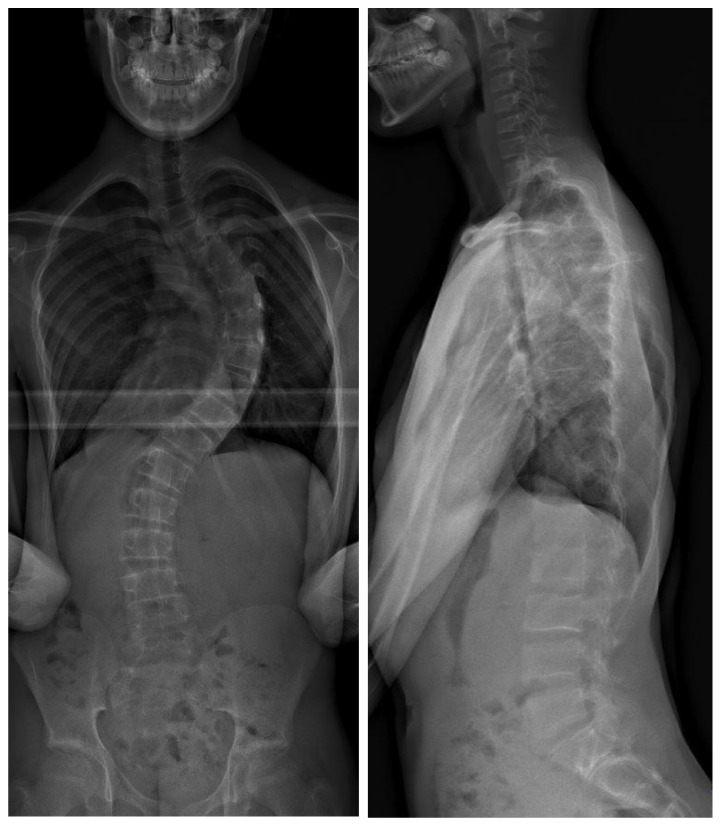
Preoperative biplanar radiograph of a 15-year-old patient with 62° thoracic scoliosis (Lenke I).

**Figure 2:**
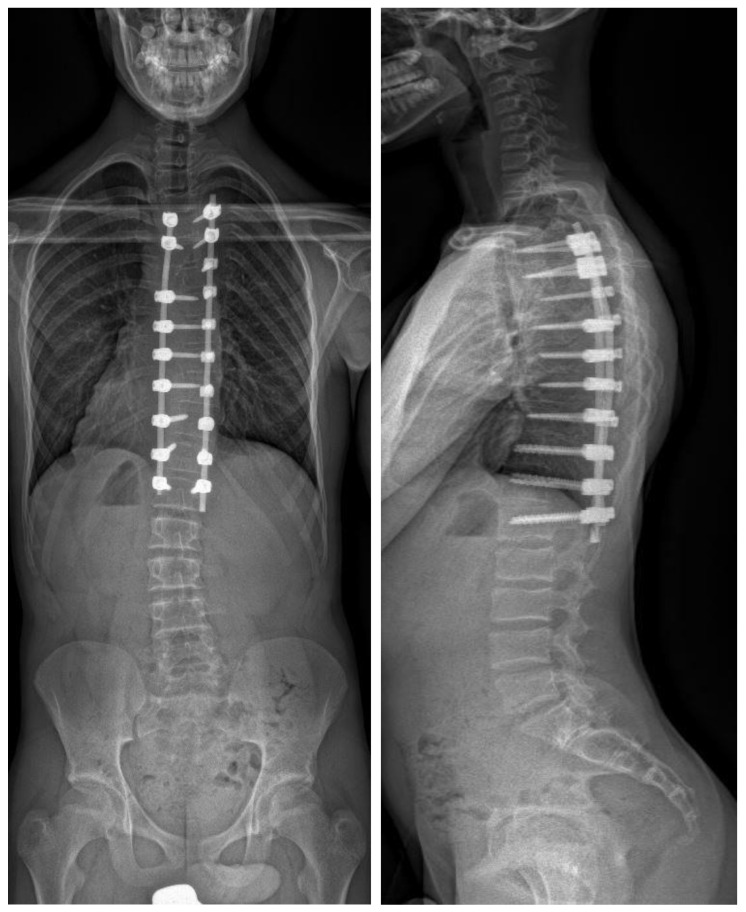
Biplanar radiograph of the same patient after posterior instrumented fusion using freehand technique. The intraoperative DAP was 638.2 mGy/cm^2^.

Freehand technique: The patient was in prone position on a radiolucent operating table (Maquet Getinge, Rastatt, Germany). After a standard posterior midline approach, entry points for pedicle screws were identified according to anatomical landmarks. Different pedicle probes were used to access the pedicles. All pedicles were tapped prior to the insertion of the pedicle screws, and the bony margins of the screw channels were checked for integrity. After the insertion of the first pedicle screw, the level was checked using fluoroscopy. The remaining pedicle screws were placed, and after placement of all pedicle screws, the implant position was checked using fluoroscopy. In the case of severe malrotation or dysplastic pedicles, additional fluoroscopies might have been necessary. After rods were inserted and deformity correction was performed, a final fluoroscopy was used to check the achieved deformity correction. During fluoroscopy, the surgical staff positioned themselves behind a mobile X-ray protective shield (WD306, Mavig GmbH, Munich, Germany). A case example is shown in Figure 1 and Figure 2.

## 3. Results

Own data on basic demographics and radiation exposure: The mean age at surgery in our cohort was 15.2 years (SD ± 1.3, range: 13–18). The mean BMI was 21.6 kg/m^2^ (SD ± 4.8). The mean follow-up was 27.4 months (range: 24–33). A total of 626 pedicle screws were implanted, corresponding to an implant density of 86.6%. In three cases, hooks were used at the upper instrumented vertebra. On average, 8.8 segments (SD ± 2.3) were fused. The mean estimated blood loss was 420 cc (SD ± 292.8) corresponding to 11.4% of estimated blood volume, and the mean surgery time was 227 min (SD ± 58.1). There were no implant-related complications. The mean Cobb angle of the main curve was 75.2° (SD ± 17.7) preoperatively and 27.7° (SD ± 10.8) postoperatively.

Table 1 provides an overview of basic demographic data, radiation exposure, and estimated lifetime attributable cancer risks of previously published studies in comparison to our cohort.

**Table 1:** FH, freehand; IN, intraoperative navigation. Values shown in italics for other studies were calculated for this study. ** *p* < 0.01; *** *p* < 0.001.

Reference (Publication Date)	Method	No. of Patients	Mean/Median Age (Years)	Mean BMI (kg/m^2^)	Mean DAP (mGy*cm^2^)	Mean DLP (mGy*cm)	Mean ED (mSv)	Estimated Lifetime Attributable Cancer Risk (%)
Dabaghi-Richerand et al. (2016) [24]	FH IN	44 37	pediatric	n/a n/a	n/a n/a	n/a n/a	0.34 (±0.36) 1.48 (±1.66) **	male: 0.0022 female: 0.0044 male: 0.0095 female: 0.019
O’Donnell et al. (2014) [25]	FH IN	43 (total)	14.2 (10–19)	n/a n/a	n/a n/a	n/a n/a	0.189 (±0.167) 7.29–9.72 (navigation) 14.58–19.44 (navigation + confirmation)	male: 0.0012 female: 0.0025 male: 0.0467–0.0622 female: 0.0948–0.126 male: 0.0933–0.124 female: 0.190–0.253
*Su* et al. *(2017) [26]*	FH IN	14 14	13 (11–18) 14 (12–18)	n/a n/a	n/a n/a	n/a n/a	0.27 (±0.2) 1.11 (±0.3) ***	male: 0.0017 female: 0.0035 male: 0.0071 female: 0.0144
Urbanski et al. (2018) [27]	FH IN	22 27	24 (12–48) 20 (11–45)	n/a n/a	n/a n/a	391 (±53) 1071 (±447) ***	6.61 (±0.90) 18.1 (±7.6) ***	male: 0.0344 female: 0.0727 male: 0.0941 female: 0.199
*Berlin* et al.* (2020) [28]*	FH	73	21.0 (±9.7)	21.5 (± 4.3)	n/a	n/a	0.17 (±0.1)	male: 0.0009 female: 0.0019
*Kapoor* et al.* (2021) [29]*	IN	4	14.3 (±1.3)	n/a	n/a	107 (±44)	2.25 (±0.8)	male: 0.0144 female: 0.0293
Own data (2021)	FH	40	15.2 (±1.3)	20.7 (±4.8)	936 (±527.8)	n/a	0.225 (±0.13)	male: 0.0014 female: 0.0029

Radiation exposure and radiogenic cancer risk: In our patient cohort, the mean DAP during surgery for AIS using the FH technique was 936.2 mGy*cm^2^ (SD ± 527.8), corresponding to an average ED of 0.225 mSv (SD ± 0.13). Table 1 provides an overview of available studies, comparing radiation dosage between surgeries for AIS with the FH technique or IN. Our average ED was within the range reported by studies using the FH technique, which showed an average ED of 1.52 mSv (SD ± 2.85). The high standard deviation is based on a strongly increased ED of 6.61 mSv from the study by Urbanski et al. [24], which was based on DLPs from 3D O-arm scans. For the studies by Dabaghi-Richerand et al. [24], Su et al. [26], O’Donnell et al. [25], and Berlin et al. [28], the mean ED is only 0.242 mSv (SD ± 0.078) and is thus more comparable to our data.

Concerning the radiation burden between surgeries for AIS with the FH technique or IN, the data show a highly significant (*p* < 0.001) increment for the mean DLP in one study (Urbanski et al.) and the mean ED available in three studies (Dabaghi-Richerand et al., Su et al., and Urbanski et al.) [24,26,27]. On average, the ED for IN was 3.7 (SD ± 0.87) times higher than for the FH technique. A deviating and up to a 51-fold increase in ED using cone-beam computed tomography IN (7.29–9.72 mSv) compared to the fluoroscopically assisted FH technique (0.189, SD ± 0.167 mSv) was reported by O’Donnell et al. [25].

The estimated lifetime attributable cancer risk was calculated from the available radiation dose data for our cohort and all the relevant literature (Table 1). According to our data, the mean ED of 0.225 mSv of the FH technique resulted in a radiogenic cancer risk of 0.0014% for males and 0.0029% for females.

These estimates are within the range of calculated data from literature values of radiation doses with an average lifetime attributable cancer risk of 0.0009–0.0344% for male patients and 0.0019–0.0727% for female patients. The respective risk projections for IN based on the relevant literature data showed significantly elevated lifetime attributable cancer risks of 0.0071–0.124% (*p* = 0.023) for male patients and 0.0144–0.253% (*p* = 0.031) for female patients, as shown in Table 1.

## 4. Discussion

The use of IN for the surgical treatment of AIS may improve the accuracy of pedicle screw placement compared to the FH technique, but it also increases radiation exposure of the patient and thus the risk for adverse late effects, including radiation-associated malignancies. Attempts have been made to reduce radiation exposure during IN by optimizing the imaging protocol, but the ED could not be reduced to a level comparable to the FH technique [30]. Our statement refers to the comparison of the IN versus the FH technique, while Su et al. compared the reduction in the effective dose by three different protocols for IN. Although the pediatric protocol of the IN measure by Su et al. [26] has reduced the effective dose to 0.65 mSv and thus to 1/4 of the average annual background dose of 2.4 mSv, this is still considerably higher compared to the vast majority of studies using the FH technique (see Table 1).

The question arises whether an improved—but clinically irrelevant—accuracy of pedicle screws using IN justifies a higher radiation burden for AIS patients [31,32]. In a recent meta-analysis, Chan et al. concluded that there is “moderate evidence of decreased breaches with CT-based IN compared with FH methods. Complication rates remain unknown due to the low complication rates from small sample sizes” [32]. Another meta-analysis by Baldwin et al. could show that pedicle screw placement is more accurate using IN compared to the FH technique. They also analyzed four studies that compared radiation exposure and found that “three out of four studies showed a significant difference in radiation exposure during CT navigation which favored freehand” technique. The fourth study showed “no difference in radiation in patients with smaller curves but showed significantly lower radiation using CT navigation when the starting curve was 74.0 degrees or higher”. However, ED just tended to be higher in IN compared to FH without reaching significance [33]. A clinically relevant reduction in complications using IN compared to the FH technique could not be identified.

We could identify only one study that showed a higher return to the operating room for the FH technique as compared to IN [34]. However, only 49% of the patients in this study had AIS; the remainder had neuromuscular scoliosis, tumor, congenital scoliosis, or other diagnoses. There is a paucity of literature directly comparing the FH technique and IN for AIS in this regard. The previously mentioned meta-analysis by Chan et al. also found that “High-risk breach rates do not have a significant difference between FH technique and image guidance methods” [32]. So far, the only studies available are those that compared radiation doses (as the DAP, DLP, or ED) for AIS patients between the FH technique or IN. A direct comparison of radiogenic cancer risk estimates as a function of age and gender has not been conducted yet for this particular patient population.

Therefore, we estimated the attributable lifetime cancer risk for our and all available studies of interest with the appropriate age- and gender-related parameters. The attributable lifetime risk for radiation-induced cancer increases with a decrease in age at exposure and can vary by a factor of more than 9 between pediatric or adolescent and elderly patients. Adolescents have the highest risks compared to children, adults, or seniors, although there is a gender-dependent difference [18,35]. For radiation exposure to the chest region, the risk of radiogenic cancer is significantly increased for women compared to men, and this is mainly due to the high risk of breast cancer [22].

The ED that we calculated for the study by Urbanski et al. [27] based on the DLP and the conversion factor for lumbar spine examinations provided by Lee et al. [23] resulted in an elevated ED of 6.61 mSv, which is within the range of the ED of a chest CT. Kapoor et al. used a comparable conversion factor to determine the ED from the DLP [35]. The mean ED of IN techniques from the literature spanned a wide range from 1.11 to 18.1 mSV and was significantly elevated compared to the mean ED of the FH technique.

The highest value of 18.1 mSv was also obtained for the study by Urbanski et al. [27], which was consistent with the ED from the study by O’Donnell et al. [25] with a maximum of 19.44 mSv for IN, including an intraoperative confirmation scan for implant positioning, and it was again consistent with the conversion factor of Kapoor et al. [29].

The gender- and age-specific risks for radiogenic cancer after PSF for AIS determined in this work differed significantly between the FH technique and IN. The corresponding average lifetime attributable cancer risk was 0.0070 (SD ± 0.013) for male patients and 0.0199 (SD ± 0.029) for female patients using the FH technique and 0.056 (SD ± 0.045, *p* = 0.023) for male patients and 0.116 (SD ± 0.092) for female patients using IN.

So far, only Perisinakis et al. aimed to determine the radiogenic risk for fatal cancer after scoliosis surgery using an anthropomorphic phantom [36]. However, the authors did not use the FH technique or IN but a fluoroscopically assisted technique for pedicle screw placement, which resulted in an average ED of 2.92 mSv for a mean of 4.8 pedicle screws. Because of the different techniques used for pedicle screw instrumentation, the study is not comparable. In our cohort, using the FH technique, the mean ED was 0.225 mSv (SD ± 0.13) for the whole surgery. The high cumulative number of radiological exposures during the treatment course of AIS further increases the risk for radiation-associated late sequelae. O’Donnell et al. demonstrated a doubling of the ED and the associated radiogenic cancer risk caused by a confirmation scan after PSF using IN [25]. Furthermore, to provide sufficient navigation data for the whole surgery, more than one scan might be necessary. In their study on pedicle perforation rates using cone-beam navigation, Oba et al. needed an average of 2.0 (SD ± 0.5, range: 1–3) scans to obtain the necessary navigation data [13]. Since the current model for estimating radiation-associated cancer risk is based on a linear dose–response relationship with no threshold, each additional scan doubles the risk of developing cancer.

The frequent diagnostic exposures of AIS patients to ionizing radiation are associated with an elevated incidence of various tumor entities, such as breast and endometrial cancer [16,19,20,37]. Law et al. evaluated the cumulative ED and the related cancer risk for scoliosis patients undergoing repetitive full spine radiography during their diagnosis and follow-up [35]. For patients exposed annually at an age between 5 and 30 years using digital radiography systems for full spine posteroanterior radiography, the authors calculated a cumulative effective dose of 15 mSv and a corresponding cumulative lifetime attributable cancer risk of 0.1–0.2%, with a significantly higher risk for women. In a Danish cohort of 215 consecutive AIS patients with a mean long-term follow-up of 24.5 years, the overall cancer rate was as high as 4.3%, with a relative risk of 4.8 for developing cancer compared to the normal age-matched Danish population [16].

We did not find any implant-related complications in our cohort. So far, one study has shown a reduction in pedicle screw misplacement using IN during surgery for AIS compared to the FH technique [33]. However, blood loss and the duration of surgery did not change significantly when using IN. Therefore, the use of IN would not have provided a clinical benefit for this group of AIS patients, but the use of IN increases radiation exposure and potentially the lifetime radiogenic cancer risk compared to the FH technique. This should be considered when using IN during surgery for AIS.

Limitations: Since a clinical benefit for using IN in AIS surgeries has not been shown yet, we did not include a control group in which IN has been used due to concerns about excessive radiation exposure. Therefore, we used previously published data on radiation exposure from IN to calculate and compare lifetime attributable cancer risk. Also, our mean follow-up of 27.4 months is relatively short.

## 5. Conclusions

IN technologies are of great benefit to the patient (and the surgeon). They may help reduce blood loss and tissue trauma in minimally invasive surgeries and help reduce the risk of relevant implant malposition in complex anatomic conditions (e.g., severe deformities or revision cases). However, for a “standard” or “straight-forward” AIS case, the use of intraoperative image-guided navigation should be considered carefully since its application significantly increases the lifetime attributable cancer risk, especially considering that AIS patients are already exposed to an increased radiation dose preoperatively and during follow-up. The use of IN may be limited to scenarios or anatomical areas that pose the most difficulty in pedicle screw placement, such as cases of congenital deformities, revision surgeries, or severely dysplastic pedicles.

## Data Availability

The data presented in this study are available on request from the corresponding author. The data are not publicly available due to data protection.
